# Exploring emotional dynamics: Word count effects in Facebook posts by sports shoe brands

**DOI:** 10.1016/j.heliyon.2024.e39808

**Published:** 2024-10-24

**Authors:** Md Shamim Hossain, Md. Asaduzzaman Babu, Kazi Md. Yusuf

**Affiliations:** Department of Marketing, Hajee Mohammad Danesh Science and Technology University, Dinajpur-5200, Bangladesh

**Keywords:** Sports shoe brands, Emotional dynamics, Facebook posts, Machine learning, COVID-19

## Abstract

This study examines the impact of word count in Facebook posts on user engagement and emotional responses, focusing on sports shoe brands. It addresses a research gap concerning the effects of post length on various emotional aspects and engagement metrics within social media marketing. Data were collected from January 1, 2024, to January 30, 2024, covering posts from January 2013 to December 2023 on official Facebook pages of several sports shoe brands. After cleaning an initial 723 posts, 636 were analyzed using statistical methods and Python libraries. Eight emotional aspects—fear, anger, sadness, trust, anticipation, joy, disgust, and surprise—were examined in relation to post length. Mean reaction values and emotional aspects were compared before and during the COVID-19 period. A structured equation model (SEM) in AMOS assessed these relationships. The analysis revealed that longer posts positively influenced trust (β = 0.208, ρ < 0.001), anticipation (β = 0.095, ρ < 0.05), joy (β = 0.259, ρ < 0.001), and surprise (β = 0.072, ρ < 0.05), but did not significantly affect fear (β = 0.065, ρ = 0.089), sadness (β = 0.054, ρ = 0.155), or disgust (β = 0.072, ρ = 0.116). During COVID-19, increased trust and anticipation were noted, while sadness and anger decreased. This study highlights that longer Facebook posts can enhance engagement through increased trust, anticipation, joy, and surprise, offering valuable insights for optimizing social media content strategies.

## 1 Introduction

Facebook, Twitter, and Reddit make it effortless for people to connect by sharing thoughts and opinions, which transmits information across society. Users can post text, photographs, and videos and respond by sharing and commenting on them, spreading information [1]. Social media is used by ordinary people for sharing and engaging with content on various subjects for entertainment, social, political, research, and economic purposes. Marketers also use it due to the sharp rise in internet users, which has inspired businesses to seek reliable ways of managing their digital footprints. Social media has achieved substantial popularity, surpassing previous predictions [2]. Facebook marketing is a common strategy that brands employ to attract customers, reach a large audience, and quickly satisfy their needs through interactive conversations [3]. This approach, commonly known as digital marketing, is an accepted strategy that enhances the impact and efficiency of marketing campaigns [4]. The sportswear industry experienced significant success in 2022, as consumer sentiment continued to rise, resulting in the easing of COVID-19 restrictions in most countries. Consequently, companies took preemptive measures by placing large orders to satisfy the growing demand and address supply chain challenges that arose in 2021 [5,6]. Sports brands embrace social media (Facebook) as a commercial and collaborative tool to augment communication, amplify brand recognition, and promote product acquisitions [7]. The seamless integration of sports into esports during the global pandemic, fueled by the audience's insatiable appetite for recreation while constrained to their homes [8], has resulted in the differentiation of sports brand positioning based on authentic market variables such as advertising and quality [9]. The sentiment of Facebook posts is a crucial indicator when studying how Facebook users evaluate a certain post and its content. Users had substantially positive emotional and logical attitudes towards social marketing and advertising communications (SMACs) that were disseminated by fellow users, as opposed to those explicitly endorsed by marketers [10]. Additionally, the components of text in branded Facebook image postings are connected to consumer engagement and brand awareness [11]. The features of content (quality, valence, and volume) impact both marketer-generated content (MGC) and user-generated content (UGC) as potential variables influencing brand equity [12].

Considering this scenario, our present investigation aims to assess the sentiments expressed in Facebook posts about sports brands by constructing machine learning models using the Emotion Wheel Theory. Sentiment analysis involves the computational examination of individuals' viewpoints, sentiments, emotional states, temperaments, and dispositions [13]. The evaluation of Facebook users’ reactions to specific content can be effectively analyzed by considering the mood expressed in their Facebook posts [14]. Sentiment analysis is utilized in various fields such as education [15], politics [16], and news [17]. Additionally, Asur & Huberman [18] used tweets to forecast box office revenue for movies.

**Fig. 1:**
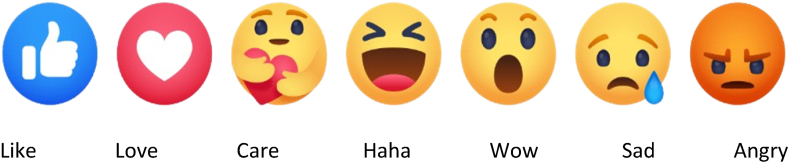
Emoji reactions on Facebook.

To the best of our knowledge, this is one of the few studies examining the emotional impact of post length and content on Facebook using the Emotion Wheel Theory. Plutchik [19] introduced a psychoevolutionary framework categorizing universal emotional reactions into eight fundamental emotions: anger, fear, sadness, disgust, surprise, anticipation, trust, and joy. In our study, we aimed to align these eight unique emotional aspects with seven diverse Facebook reaction categories: Like, Love, Care, Haha, Wow, Sad, and Angry (Fig. 1) [14], using machine learning techniques. Understanding Facebook users' perceptions of sports and FMCG businesses can have significant implications. Marketers and decision-makers will gain valuable insights into public sentiment regarding specific brands and products, identify potential engagement barriers, and communicate more effectively. Moreover, by addressing various types of reactions, marketers can enhance customer understanding and increase the relevance and effectiveness of their strategies. Predicting user attitudes towards products and brands serves as a powerful tool for understanding consumer behavior.

## 2 Literature review

### 2.1 Facebook marketing of sportswear brands

The term “social media” combines the meanings of the words “social” and “media.” “Social” refers to interactions between groups of people who share interests or between members of the same community, while “media” refers to any medium that facilitates the dissemination and consumption of user-generated content [20]. In the past decade, social media platforms such as Facebook, Instagram, and WhatsApp have experienced massive user growth [21]. The numerous social and emotional benefits of participating in social media may explain its widespread use [22]. Recent studies have highlighted several key factors for enhancing social media marketing effectiveness. Singh [23] identifies critical issues such as customer support, platform trust, secure data sharing, and perceived value, which significantly impact social media marketing success. Maduku [24] explores how top management participation influences social media assimilation in B2B firms, finding that assimilation into channel management positively affects sales performance and relationship development, though its impact varies in other areas. Additionally, Mulcahy et al. [25] demonstrate that self-gain framed content significantly enhances believability, which in turn improves engagement and purchase intentions. These insights collectively underscore the importance of addressing specific challenges, management involvement, and effective message framing to optimize social media marketing outcomes.

An increasing number of organizations are incorporating social media marketing into their business plans [26]. Social media marketing tools have grown in importance, allowing marketers to better understand their customers, build relationships, and ultimately increase sales, brand recognition, and product quality [27]. Effective social media marketing not only enhances brand recognition but also facilitates conversations between marketers and consumers [28]. Identifying advertising targets on social networks has become a significant concern in strategic planning. With ad targeting, businesses can profile their target audience more effectively and determine the sequence of their marketing efforts. Facebook, the largest social networking site with over 1.79 billion users globally, offers a wide variety of advertising targets [29]. With 3.05 billion monthly active users, excluding family services like WhatsApp, Messenger, and Instagram, and an estimated 5.3 billion internet users worldwide [30], there are now more ways than ever to reach large audiences. The rise of digital platforms has profoundly impacted the sports sector [31]. Factors related to Facebook posts and individual user experiences, including information overload, usage patterns, and various user conditions, contribute to both passive and active participation on these sites [32]. Social media enables sports organizations, athletes, coaches, fans, and partners to reach global audiences and build, expand, retain, and enhance brand equity through ongoing communication. Sport marketers focus on brand management and developing content that boosts brand image and equity [33]. Research shows that social media marketing capabilities positively affect marketing performance through a mediating effect on social media marketing performance [34].

### 2.2 Facebook content and emotion wheel theory

Emotional messages often prove more effective than informative ones in certain situations, and several guidelines for Facebook usage have been developed for managing destinations via social media [35]. The popularity of “just for fun” posts is unsurprising to users. It is assumed that consumers are more inclined to engage with cheerful and personable content rather than formal and technical articles [36]. Posts are generally more engaging and popular when they contain rich content and are relatively brief. Negative reactions tend to have a stronger impact on engagement than positive ones, except in cases of sharing. However, statistical analysis has indicated that the timing of a post has minimal influence on its interaction or popularity [37]. Currently, Facebook allows the categorization of audience sentiment through various reactions, such as positive ones (love, care, laughter, and wow) and negative ones (sad and angry) [38].

The increasing prevalence of social media data presents an appealing opportunity to enhance the quality of machine learning-based models across various domains, including computer vision and natural language processing. Research explores the correlations between different classifications of emotions commonly considered in detecting emotions in Facebook posts and the “reactions” of numerous individuals [39]. Online social media users' responses to content are influenced by the surrounding context, with emotions or moods significantly affecting these reactions and leading to the spread of opinionated information on these platforms [40].

Behavioral scientists studying emotions have long grappled with how to measure them comprehensively and meaningfully. Due to their subjective nature and the higher-level semantics that are difficult to quantify scientifically, there is considerable debate about the emotions and affects expressed in artwork [41]. The natural language processing approach to sentiment analysis extracts sentimental, opinion, or emotional information from text data. Sentiment analysis in natural language processing is inherently challenging and resource-intensive [42]. This publication presents a Plutchik wheel of emotion and a hybrid machine learning-based sentiment analysis approach to address these challenges. Psychological research has established strong correlations among human emotions, with certain emotions frequently co-occurring and showing positive links, while others are inversely related. The emotion wheel theory, introduced in 1980, provides a well-known framework for explaining these interconnections. This model identifies eight fundamental emotions: anger, contempt, sadness, surprise, fear, trust, joy, and anticipation. The EWA-EDL model calculates correlations between these fundamental emotions using this framework and integrates psychological prior information into a multi-task convolutional neural network with an attention mechanism [43]. Sentiment analysis aims to predict the sentiment of words, sentences, or documents. This study investigates sentiment polarization on Twitter by categorizing sentiments using Plutchik's wheel of emotions, which simplifies analysis through eight fundamental emotions: anger, fear, sadness, disgust, surprise, anticipation, trust, and joy. This approach is supported by several relevant studies. Grădinaru et al. [44] explore how sustainability influences brand attachment and consumer behavior, offering a framework for understanding the effect of eco-friendly attributes on sentiment towards brands. Musova et al. [45] examine consumer attitudes towards circular models in fashion, highlighting the impact of sustainability on consumer support and engagement, which can parallel user responses to sustainable sports shoe brands. Obadă and Dabija [46] investigate how social media users share fake news about environmentally friendly brands, providing insights into how misinformation might skew sentiment analysis results. Kumar and Vardhan [47] utilize Plutchik's wheel for sentiment analysis, demonstrating its effectiveness in addressing sentiment polarization. Bolioli et al. [48] apply Plutchik's theory to emotion extraction in cultural heritage, showcasing the utility of the emotional framework in diverse contexts. Borghi and Mariani [49] examine the role of emotions in interactions with social robots, while Saraff and Tripathi [50] explore the link between emotional intelligence and facial expression recognition, contributing to a broader understanding of emotional context in sentiment analysis. Lee and Kim [51] review basic emotion theories, providing a critical assessment of Plutchik's model in comparison with other theories. Kumar and Vardhan [42] further extend this work by applying a hybrid sentiment analysis approach for Hindi, illustrating the adaptability of Plutchik's model in different linguistic and computational settings.

We collected data from the official Facebook pages of ten popular shoe brands: Adidas, Asics, Columbia Sportswear, New Balance, Nike, Puma, Reebok, and Under Armour. The analysis was conducted using Plutchik's wheel of emotions as a theoretical framework, including the eight primary human emotions: anger, fear, anticipation, trust, surprise, sadness, joy, and disgust [52]. A study found that fine-tuning a language model with emotional values of words improved its effectiveness [51]. This research focused on Facebook pages of the aforementioned shoe brands.

### 2.3 Facebook post length and emotional aspects

Recent research underscores the significant role of post length and emotional content in shaping user engagement on social media. Liu et al. [53] introduce a Deep Parallel Contextual Analysis Framework (DPCAF) to enhance emotion prediction in social media texts, showing that detailed semantic analysis improves emotion detection, particularly for short texts. Their findings highlight the importance of comprehensive textual analysis in capturing nuanced emotional responses. Yan et al. [54] developed an emotion-information multiplex network that illustrates how emotion influences information propagation on social media, revealing that emotional content significantly impacts user engagement and interaction. Kodati and Dasari [55] employ advanced deep learning techniques to detect a wide range of emotions in social media, demonstrating that detailed emotional insights can be derived from contextually rich content. Zhao et al. [56] find that emotional content, particularly negative emotions, enhances user engagement behaviors on Facebook, suggesting that the emotional primacy effect can be leveraged by varying post lengths and emotional tones. This body of work collectively supports the hypothesis that longer Facebook posts with rich emotional content positively influence user engagement with sports shoe brands.

The utilization of social media platforms, such as Facebook, can elicit both favorable and unfavorable emotions, and the findings of previous research on the psychological impacts of social media usage are heterogeneous [53,54,[57], [58], [59]]. Liu et al. [53] highlight the challenges in emotion prediction on social media due to the brevity and semantic limitations of short texts. Yan et al. [54] emphasized how emotions influence the spread of information and impact public sentiment. Scotland et al. [59] further demonstrate the varied emotional responses to significant sociopolitical events on social media, reflecting the heterogeneous nature of public sentiment. This reinforces the notion that social media platforms provoke a wide range of emotional reactions. Numerous studies have provided evidence that social media platforms, such as Facebook, facilitate the transmission of emotional material through social channels. These platforms enhance the visibility and likelihood of public sharing of corporate posts that evoke strong emotions [60]. Users often report more positive than negative feelings after using Facebook [61]. The use of concise writing results in a limited range of features [62], and findings show that the most common feelings conveyed are happiness, excitement, and trust. Multivariate regression analysis indicates that joy has a greater influence on customers compared to surprise and anticipation [49]. The presence of rich content, such as images and videos, increases engagement, while comments are affected by images and publishing timing [63]. A positive correlation is observed between longer content and engagement, while high post frequency and early morning posting show negative correlations [64]. Based on this literature, the study hypothesizes.

H1 Word count of Facebook post has a positive influence of emotional aspects towards Sports Shoe Brands.

## 3 Methodology of the study

Between January 1, 2024, and January 30, 2024, we collected data on posts made from January 2013 to December 2023 on the official Facebook pages of various shoe brands. Our dataset initially comprised 723 posts from brands such as Adidas, Asics, Columbia Sportswear, Heinz, Lays, New Balance, Nike, Puma, Reebok, and Under Armour. Each post included details on its content and the number of reactions from readers. We then cleaned the data to remove any missing values, resulting in 636 posts available for further analysis. For the analysis, we used both descriptive and inferential statistical techniques. We utilized the Python programming language along with libraries such as Pandas, NumPy, Matplotlib, Seaborn, and NLTK to identify patterns, trends, and correlations in the dataset. A key focus of our study was emotional analysis, where we examined eight distinct emotional aspects for each post: fear, anger, sadness, trust, anticipation, joy, disgust, and surprise. Additionally, we calculated the length of each post by counting the number of words. This analysis aimed to uncover potential relationships between post length and user engagement or emotional responses.

We also compared the mean values of reactions and emotional aspects before and during the COVID-19 pandemic to assess potential shifts in user sentiments over time. For a deeper exploration of the relationships between post length and emotional aspects, we employed AMOS (Version 24) to perform a Structural Equation Modeling (SEM) analysis. The SEM analysis provided estimates (β) and critical ratios (C.R.) for each hypothesized path, offering insights into the strength and significance of these relationships. Overall, our methodological approach combined data collection, cleaning, analysis, and visualization techniques to conduct a comprehensive study of user reactions and emotional responses to Facebook posts from various shoe brands. This approach allowed us to gain valuable insights into the influence of post length and content on user engagement and emotions.

## 4 Analysis and result discussion

Table 1 presents quantitative data on the frequency of Facebook posts made by various sports brands to promote their products. Columbia Sportswear led with the highest number of posts, totaling 141, and had an average post length of 214.08 words. In comparison, Asics had fewer posts than Columbia Sportswear but had the highest average post length at 258.15 words. Reebok, on the other hand, had the fewest posts and an average post length of 189.83 words, ranking third among the ten brands for average word count. The study encompasses a total of 636 posts related to sportswear brands during the COVID-19 period.

**Table 1:** Number of Facebook posts and average number of words used in posts towards different brands.

Brand name	Post count	Average Word count
Adidas	78	167.59
Asics	133	258.15
Columbia Sportswear	141	214.08
Heinz	31	114.26
Lays	76	57.61
New Balance	66	147.12
Nike	34	66.88
Puma	71	53.21
Reebok	6	189.83
Under Armour	47	55.23
**Total**	**636**	

Table 2 illustrates the percentile representation of seven distinct reactions exhibited by Facebook users in response to posts from the sampled brands. Adidas received a predominant 90.17 % positive reactions and only 0.05 % negative reactions. Columbia Sportswear garnered 97.97 % positive reactions and reported no sad reactions. Asics had 90.58 % likes and 0.03 % unhappy reactions. Heinz and Lays both recorded high positive reactions, with Heinz at 71.13 % and Lays at 65.80 %. For Heinz, only 0.20 % of reactions were sad, while Lays had 0.45 % angry reactions. New Balance, Nike, and Puma showed strong positive reactions, with shares of 90.42 %, 90.54 %, and 80.93 % respectively. These brands also had low percentages of sad reactions, with New Balance at 0.02 %, Nike at 0.07 %, and Puma at 0.03 %. Reebok and Under Armour were the top brands in terms of likes, with 86.78 % and 87.83 % respectively, and they also had very low percentages of sad emotions, at 0.02 % and 0.05 % respectively.

**Table 2:** Percentage distribution of Facebook reactions towards each brand.

Brand name	Angry	Care	Haha	Like	Love	Sad	WOW	Total
Adidas	0.25 %	0.20 %	0.95 %	90.17 %	7.71 %	0.05 %	0.67 %	100.00 %
Asics	0.47 %	0.29 %	0.07 %	90.58 %	8.22 %	0.03 %	0.36 %	100.00 %
Columbia Sportswear	0.14 %	0.09 %	0.14 %	97.97 %	1.58 %	0.00 %	0.07 %	100.00 %
Heinz	0.68 %	0.67 %	16.75 %	71.13 %	9.59 %	0.20 %	0.98 %	100.00 %
Lays	0.45 %	0.80 %	6.56 %	65.80 %	13.07 %	0.50 %	12.81 %	100.00 %
New Balance	0.11 %	0.06 %	0.53 %	90.42 %	7.56 %	0.02 %	1.29 %	100.00 %
Nike	0.35 %	0.24 %	0.21 %	90.54 %	8.10 %	0.07 %	0.48 %	100.00 %
Puma	0.03 %	0.56 %	0.32 %	80.93 %	16.00 %	0.13 %	2.03 %	100.00 %
Reebok	0.37 %	0.51 %	0.15 %	86.78 %	11.66 %	0.02 %	0.51 %	100.00 %
Under Armour	0.07 %	0.67 %	0.34 %	87.83 %	10.08 %	0.05 %	0.95 %	100.00 %

Table 3 presents average values of reactions towards Facebook posts, highlighting the highest and lowest values for each brand, which provide key insights. Adidas posts receive a substantial number of positive “Like” reactions, averaging 2983.128, the highest among the brands, while the average number of “Sad” reactions is 1.654, indicating that Adidas posts elicit minimal sadness. Asics also shows strong positive engagement with an average of 193.526 likes, and a very low average of 0.060 sad reactions, suggesting that Asics' content does not significantly upset users. Columbia Sportswear has an average of 431.582 likes, reflecting solid engagement, and a low average of 0.021 sad reactions. Heinz's content generates 46.677 ″Haha” reactions, indicating that it is amusing, while the average number of sad reactions is 0.548. Lays garners 153.289 ″Wow” reactions, suggesting that its content surprises or amazes users, though it also has 6.000 sad reactions, indicating that it is not particularly saddening. New Balance averages 443.778 likes and 0.121 sad reactions, indicating that its content is largely well-received and not distressing. Nike stands out with an exceptionally high average of 16,835.294 likes, reflecting a massive positive response. Puma also shows positive engagement with an average of 2560.225 likes and low sad reactions at 4.056. In contrast, Under Armour has a remarkably high average of 22,436.106 likes with relatively low angry reactions at 19.085. Reebok, while having a respectable average of 853.667 likes and low sad reactions at 0.167, does not evoke significant sadness in users compared to other brands.

**Table 3:** Average value of reactions towards the Facebook posts for each brand.

Brand name	Like	Love	Care	Haha	WOW	Sad	Angry
Adidas	2983.128	255.154	6.526	31.436	22.064	1.654	8.410
Asics	193.526	17.556	0.609	0.143	0.767	0.060	1.000
Columbia Sportswear	431.582	6.957	0.383	0.624	0.312	0.021	0.624
Heinz	198.194	26.710	1.871	46.677	2.742	0.548	1.903
Lays	787.145	156.408	9.526	78.447	153.289	6.000	5.421
New Balance	443.788	37.121	0.288	2.591	6.348	0.121	0.530
Nike	16835.294	1506.941	45.294	39.559	88.794	13.676	64.706
Puma	2560.225	506.113	17.831	10.127	64.324	4.056	0.845
Reebok	853.667	114.667	5.000	1.500	5.000	0.167	3.667
Under Armour	22436.106	2575.128	172.085	87.191	241.660	13.617	19.085

Table 4 presents the mean values of reactions before and during the COVID-19 period, highlighting notable variations in user engagement across different reaction types. Before the COVID-19 period, the “Like” reaction had the highest mean value of 4849.503, indicating that users predominantly expressed positive sentiments through likes. In contrast, the “Care” reaction had the lowest mean value of 7.893, suggesting it was less frequently used by users. During the COVID-19 period, the “Like” reaction maintained the highest mean value at 2707.443, reflecting continued strong positive sentiments. Conversely, the “Angry” reaction had the lowest average value of 4.634 during this period, indicating a relatively lower expression of anger compared to other reactions.

**Table 4:** Mean value of reactions towards COVID-19 period.

COVID period	Like	Love	Care	Haha	WOW	Sad	Angry
Before COVID - 19	4849.503	377.887	7.893	10.960	40.938	2.847	12.525
During COVID - 19	2707.443	356.494	21.684	28.399	50.887	2.986	4.634

Table 5 shows the mean emotional values before and during COVID-19. Fear slightly increased during the pandemic to 0.021, while anger peaked earlier at 0.023 and decreased during the pandemic to 0.018. Sadness (depression) had a higher mean before COVID-19 at 0.021 compared to 0.011 during the pandemic. Trust and anticipation both grew during COVID-19, with mean values of 0.098 and 0.125, respectively, exceeding their pre-pandemic levels. Joy rose from 0.030 before the pandemic to 0.070 during it. Disgust increased more during COVID-19, with a mean value of 0.005 compared to 0.001 before the pandemic. Finally, the pandemic led to an increase in the mean value of surprise, which rose to 0.033 from 0.020. These findings illuminate the complex emotional responses during COVID-19, contributing to academic research on the pandemic's psychological effects.

**Table 5:** Mean value of emotional aspects towards COVID -19 period.

COVID period	fear	anger	sadness	trust	anticipation	joy	disgust	surprise
**Before COVID - 19**	0.019	0.023	0.021	0.049	0.069	0.030	0.001	0.020
**During COVID - 19**	0.021	0.018	0.011	0.098	0.125	0.070	0.005	0.033

Initially, the SEM analysis revealed that the model fit values were not significant. Consequently, we excluded the anger variable and concentrated on the remaining seven emotional aspects, leading to improved model fit values. Table 6 shows the results of a regression study examining the relationship between word count and various emotional states (Fear, Sadness, Trust, Anticipation, Joy, Disgust, and Surprise). The structured equation model demonstrates a good fit, as the model fit indices are within the acceptable range: CMIN/df < 3, GFI ≥0.80, AGFI ≥0.80, CFI ≥0.90, RMSEA ≤0.08 [[65], [66], [67]]. The model fit indices fall within this range, indicating a perfect fit. All emotional aspect estimates (β) are positive, suggesting that longer word counts in Facebook posts have a positive impact on emotional responses. The findings support significant positive impacts on Trust (β = 0.208, p < 0.001), Anticipation (β = 0.095, p < 0.05), Joy (β = 0.259, p < 0.001), and Surprise (β = 0.072, p < 0.05). However, the model did not support Fear (β = 0.065, p = 0.089), Sadness (β = 0.054, p = 0.155), and Disgust (β = 0.072, p = 0.116).

**Table 6:** Path analysis (summary of the result).

Hypothesized Paths			Estimates (β)	C.R.	ρ	Result
Word Count	→	Fear	0.065	1.700	0.089	Not Supported
Word Count	→	Sadness	0.054	1.423	0.155	Not Supported
Word Count	→	Trust	0.208	5.557	∗∗∗	Supported
Word Count	→	Anticipation	0.095	2.493	∗∗	Supported
Word Count	→	Joy	0.259	6.996	∗∗∗	Supported
Word Count	→	Disgust	0.060	1.573	0.116	Not Supported
Word Count	→	Surprise	0.072	1.894	∗∗	Supported

Note: ∗∗∗p < 0.001, ∗∗p < 0.05. Source: SEM-Amos output.

Model Fit Indices: CMIN/df = 1.75; GFI = 0.988; AGFI = 0.977; CFI = 0.90; RMSEA = 0.033.

Furthermore, our findings align with recent research emphasizing the importance of post length and emotional content in shaping social media engagement. Liu et al. [53] highlight that comprehensive semantic analysis improves emotion detection, especially in shorter texts. Our study supports this by showing that longer posts with detailed content lead to higher user engagement, consistent with Mariani et al. [64], who found a positive correlation between post length and engagement. Yan et al. [54] demonstrate that emotional content significantly influences information propagation and user engagement, a finding corroborated by our results showing that emotionally rich posts, like those from Adidas and Columbia Sportswear, receive more positive reactions. Kodati and Dasari [55] also use advanced deep learning techniques to detect a wide range of emotions, supporting our results that detailed emotional analysis enhances user interactions.

Regarding negative emotions, Zhao et al. [56] find that emotional content, especially negative emotions, can boost user engagement. While our study also acknowledges the impact of negative emotions, it highlights that positive reactions are more prevalent and influential overall. This nuanced understanding extends Zhao et al.’s findings by showing that, although negative emotions do affect engagement, positive emotions dominate. Scotland et al. [59] document varied emotional responses to significant sociopolitical events, reflecting a heterogeneous nature of public sentiment. Our results further this by showing shifts in emotional responses during the COVID-19 pandemic, such as increased trust and anticipation and decreased sadness. This variability aligns with Sheldon et al. [57] and Grieve et al. [58], who discuss the diverse psychological impacts of social media. Our study also confirms Bazarova et al. [60]'s findings that platforms amplify the visibility and sharing of emotionally engaging content. By showing that brands with rich emotional content, such as those with extensive textual elements, achieve higher engagement, we reinforce the notion that both textual and multimedia richness enhance user interaction. This is further supported by Sabate et al. [63], who find that rich content increases engagement. Finally, our analysis of emotional responses before and during the COVID-19 pandemic shows increased trust and anticipation, alongside decreased sadness. This observation is consistent with previous research suggesting that significant global events can shift emotional responses and influence engagement patterns on social media. Overall, our findings align with and extend recent research, validating the critical role of post length and emotional content in user engagement.

## 5 Conclusion

The primary purpose of the research is to investigate the ways in which the content of Facebook has an influence on the emotional aspects. The results of the study were obtained by the application of the emotion wheel theory and the machine learning technique. Furthermore, the findings of the study make it abundantly evident that the word count of content on Facebook does, in fact, matter, and that it does have a beneficial effect on emotional aspects, despite the fact that the aspects are not statistically supported, as is noted in Table 6. Therefore, the length of a Facebook post, which is referred to as the word count, has a huge impact on trust, anticipation, joy, and surprise for the recipient. Shorter postings frequently elicit higher levels of engagement due to their expedited readability and digestibility. Concise and focused posts have the potential to effectively attract users' attention, resulting in increased rates of interaction, including likes, comments, and shares. Conversely, lengthier posts have the opportunity for more intricate narratives and the articulation of sentiments. They possess a heightened capacity to elicit intense emotional reactions from readers, since they can explore the subject matter in greater depth, recount personal tales, or offer additional context. Nevertheless, the emotional resonance is primarily contingent upon the caliber of the writing and the capacity to establish an emotional connection with the audience. In summary, the emotional impact of both short and long Facebook posts is contingent upon various aspects, including the relevancy of the information, the quality of the writing, the preferences of the audience, and the desired emotional response. The process of conducting experiments and analyzing engagement metrics can be important in determining the optimal length of posts that can effectively enhance emotional resonance with one's audience.

### 5.1 Theoretical implication

The study can offer empirical evidence for the validity and applicability of the Emotion Wheel theory in the context of social media interactions by utilizing it as a framework for assessing user replies. This study aims to illustrate the manifestation of several emotional categories as delineated in the Emotion Wheel theory through users' responses to online material. The utilization of machine learning methodologies for the identification of users' emotional reactions gives an innovative methodology for analyzing emotions within the domain of social media. This work aims to showcase the efficacy of machine learning algorithms in the automated classification and interpretation of emotions conveyed through textual data. By doing so, it seeks to offer valuable insights into the possible uses of artificial intelligence in comprehending human emotions in the online realm. A study utilizing the Emotion Wheel theory and machine learning techniques to identify users' reactions to Facebook content has the capacity to enhance our theoretical comprehension of emotions in online environments. Additionally, it can provide valuable insights for practical implementations in social media analytics and content recommendation systems.

### 5.2 Practical implication

The Emotion Wheel theory and machine learning techniques can be used to assess customer comments on Facebook and other social media platforms. Organizations have the potential to acquire more profound understandings of customers' emotional responses towards their offerings, services, and brand image, thereby empowering them to make informed choices based on data in order to enhance customer contentment and allegiance. The findings of this study have the potential to enhance product development procedures through the identification of emotional requirements and preferences within certain target demographics. Organizations can utilize this data to develop products and services that elicit an emotional response from customers, resulting in increased levels of customer satisfaction and loyalty. The work has the potential to enhance the advancement of sentiment analysis tools and algorithms by considering the intricacies of human emotions. Businesses, marketers, and researchers can utilize these technologies to analyze substantial amounts of social media data and derive significant insights for the goal of making informed decisions. A study utilizing the Emotion Wheel theory and machine learning techniques to identify people' reactions to Facebook material can have practical ramifications for a wide range of stakeholders, including marketers, advertisers, community managers, and product developers. Organizations may optimize their social media strategy, promote consumer engagement, and achieve financial success by utilizing insights about users' emotional responses.

### 5.3 Limitation and future work

While our study sheds light on the intriguing relationship between Facebook post length and emotional aspects, it's important to acknowledge its limitations and outline exciting avenues for future research. Firstly, our sample was confined to a specific time frame and a select group of sportswear brands, potentially limiting the generalizability of our findings. Moreover, focusing solely on official Facebook pages might overlook valuable insights from user-generated content and interactions, warranting further exploration. Additionally, our reliance on machine learning techniques for emotion classification, while efficient, may lack the nuanced understanding provided by human interpretation. Furthermore, the correlational nature of our analysis precludes causal inferences, prompting the need for more robust experimental designs. Looking ahead, there are numerous exciting avenues for future research to explore. Longitudinal studies could track changes in the relationship between post length and emotional engagement over time, uncovering dynamic trends and patterns. Cross-platform analyses could delve into how post length influences emotional responses across different social media platforms, enriching our understanding of user behavior in diverse contexts. Qualitative approaches, such as interviews or focus groups, could offer deeper insights into the underlying mechanisms driving user responses to Facebook content. Investigating user-generated content, such as comments and shares, would provide a more comprehensive view of emotional engagement on social media. Moreover, considering cultural differences in emotional expression and interpretation could enhance the relevance and applicability of our findings across diverse populations. Finally, experimental designs that manipulate post length in controlled settings would enable us to establish causal relationships and better understand the impact of content length on emotional responses. By addressing these limitations and embracing these exciting research directions, we can unlock a deeper understanding of how content length influences emotional engagement on social media platforms like Facebook. This, in turn, can inform strategies for content creators, marketers, and businesses to better connect with their target audiences and foster meaningful engagement online.

## CRediT authorship contribution statement

**Md. Shamim Hossain:** Writing – review & editing, Writing – original draft, Visualization, Validation, Supervision, Software, Project administration, Methodology, Investigation, Formal analysis, Conceptualization. **Md. Asaduzzaman Babu:** Writing – review & editing, Writing – original draft, Visualization, Software, Resources, Methodology, Investigation, Formal analysis, Data curation. **Kazi Md. Yusuf:** Writing – review & editing, Writing – original draft, Validation, Resources, Methodology, Investigation, Formal analysis, Conceptualization.

## Data and code availability statement

Data will be made available on request.

## Declaration of competing interest

The authors declare that they have no known competing financial interests or personal relationships that could have appeared to influence the work reported in this paper.
